# Sulfatides Partition Disabled-2 in Response to Platelet Activation

**DOI:** 10.1371/journal.pone.0008007

**Published:** 2009-11-24

**Authors:** Karen E. Drahos, John D. Welsh, Carla V. Finkielstein, Daniel G. S. Capelluto

**Affiliations:** 1 Protein Signaling Domains Laboratory, Virginia Polytechnic Institute and State University, Blacksburg, Virginia, United States of America; 2 Integrated Cellular Responses Laboratory Department of Biological Sciences, Virginia Polytechnic Institute and State University, Blacksburg, Virginia, United States of America; University of Queensland, Australia

## Abstract

**Background:**

Platelets contact each other at the site of vascular injury to stop bleeding. One negative regulator of platelet aggregation is Disabled-2 (Dab2), which is released to the extracellular surface upon platelet activation. Dab2 inhibits platelet aggregation through its phosphotyrosine-binding (PTB) domain by competing with fibrinogen for αIIbβ3 integrin receptor binding by an unknown mechanism.

**Methodology/Principal Findings:**

Using protein-lipid overlay and liposome-binding assays, we identified that the N-terminal region of Dab2, including its PTB domain (N-PTB), specifically interacts with sulfatides. Moreover, we determined that such interaction is mediated by two conserved basic motifs with a dissociation constant (*K_d_*) of 0.6 µM as estimated by surface plasmon resonance (SPR) analysis. In addition, liposome-binding assays combined with mass spectroscopy studies revealed that thrombin, a strong platelet agonist, cleaved N-PTB at a site located between the basic motifs, a region that becomes protected from thrombin cleavage when bound to sulfatides. Sulfatides on the platelet surface interact with coagulation proteins, playing a major role in haemostasis. Our results show that sulfatides recruit N-PTB to the platelet surface, sequestering it from integrin receptor binding during platelet activation. This is a transient recruitment that follows N-PTB internalization by an actin-dependent process.

**Conclusions/Significance:**

Our experimental data support a model where two pools of Dab2 co-exist at the platelet surface, in both sulfatide- and integrin receptor-bound states, and their balance controls the extent of the clotting response.

## Introduction

Platelets are anucleate cell fragments that contact each other to form a plug at the site of vascular injury. Platelets contain both α- and δ-granules that contribute to their adhesion, activation, and aggregation [1]. Granules contain a variety of proteins including adhesive and plasma proteins, coagulation and anti-coagulation factors, and protease inhibitors [2]. Platelet adhesion to a prothrombotic surface is mediated by the formation of the glycoprotein Ib-IX-V and von Willebrand factor (vWF) complex, which is followed by the release of granules and the activation of members of the integrin family of receptors. Activated platelets accelerate the coagulation process by providing the membrane surface necessary for the generation of the active form of thrombin, which acts as a potent platelet agonist (for review, see [3]). Thus, vWF initiates the formation of stable aggregates under high shear flow conditions. Other complexes, such as P-selectin-sulfatides and αIIbβ3 integrin-fibrinogen, also stabilize platelet aggregates. Thrombin cleaves fibrinogen to fibrin, which crosslinks platelets to form the haemostatic plug.

Mammals present two Dab orthologs, Dab1 and Dab2, which share an N-terminal PTB domain and a C-terminal proline-rich SH3 domain, indicating that they function as adaptor proteins [4]. Dab1 is almost exclusively expressed in the brain [5], whereas Dab2 expression is widely distributed [6]. Dab2 has been implicated in growth factor signaling [7], endocytosis [8], inhibition of platelet aggregation [9], and is known to interfere with the Wnt signaling pathway [10]. Expression of Dab2 is frequently lost in 85–90% of breast and ovarian carcinomas and homozygous deletions of its gene have been identified in a small percentage of tumors [6], [11]. Downregulation of Dab2 has been identified in a disparity of cancers including esophageal [12] and prostate carcinomas [13] and found associated to early stage events [14]. These observations led to the hypothesis that Dab2 is a tumor suppressor.

The PTB domain is evolutionarily conserved and often found in proteins containing other protein-protein interaction domains. This module was initially recognized to bind tyrosine-phosphorylated (pTyr) NPXY motifs [15]. However, the Dab2 PTB domain interacts with several non-phosphorylated NPXY-containing proteins, including the low-density lipoprotein (LDL) receptor protein 1 [8] and Dishevelled-3 [10]. The Dab2 PTB domain plays a key role in the LDL receptor internalization as Dab2 co-localizes with clathrin coats on the cell membrane during endocytosis [8], [16]. In Dab1, the PTB domain preferentially binds to phosphatidylinositol 4,5-biphosphate (PtdIns(4,5)P_2_) [16], an interaction that has been proposed to target Dab1 to membranes [17]. In Dab2, the PTB domain mediates binding of the protein to both the lipoprotein receptor and PtdIns(4,5)P_2_ during endocytosis [16]. In addition, the PTB domain mediates binding of Dab2 to the αIIbβ3 integrin receptor, thus, exerting its negative role in platelet aggregation [9] by an unknown mechanism.

Sulfatides, the sulfuric ester of galactosylceramides at the C3 of the galactose residue, are present at the cell surface and are thought to influence the activity of integral membrane proteins [18]. Sulfatides are known to participate in cell adhesion, differentiation and signal transduction [19]. This sphingolipid, which increases its levels upon platelet activation [20], has been shown to bind coagulation proteins including vWF [21], P-selectin [22], and thrombospondin [23], thus, playing a key role in haemostasis. Here, we have specifically investigated the mechanism by which Dab2 regulates platelet function. We found that the N-terminal region of Dab2 specifically binds sulfatides through two conserved polybasic motifs, and this association partitions the protein into two pools at the platelet surface, the sulfatide- and integrin receptor-bound states, thus, modulating the extent of platelet activation.

## Methods

### DNA Cloning, Plasmids, and Protein Expression and Purification

The Dab2 N-PTB (residues 1-241) cDNA was cloned into pGEX6P1 (GE Healthcare). Site-directed mutagenesis of Dab2 N-PTB was performed using Quick Change (Stratagene). Proteins were expressed in *E.coli* Rosetta and purified on glutathione beads as described [24]. Purity of all proteins was over 95% as judged by SDS-PAGE.

### Protein-Lipid Overlay Assay

Membrane strips (Echelon Research Laboratories) spotted with 100 pmoles of sphingolipids were incubated with 0.1 µg/ml of N-PTB or its mutants in 20 mM Tris-HCl (pH 8.0), 150 mM NaCl, 0.1% Tween-20, and 3% fatty acid-free BSA overnight at 4°C. Following four washes with the same buffer, bound proteins were probed with rabbit anti-GST antibody (Santa Cruz Biotech) and donkey anti rabbit-horse radish peroxidase (HRP) (GE Healthcare). Protein binding was detected using ECL reagent (Pierce). Lipid strips were prepared by spotting 1 µl of the indicated amount of either sulfatide or PtdIns(4,5)P_2_ dissolved in chloroform:methanol:water (1∶2∶0.8 and 65∶35∶8, respectively) onto Hybond-C extra membranes (GE Healthcare) and protein binding was monitored as described above.

### Liposome-Binding Assay

Stocks of brain sulfatides, phosphatidylcholine (PC), phosphatidylethanolamine (PE), phophatidylserine (PS) (Avanti Polar Lipids), cholesterol (Sigma) and PtdIns(4,5)P_2_ (Cayman Chemicals) were prepared in organic solvents per manufacturer instructions. Sulfatide liposomes were prepared by weight ratio of 1∶1∶1∶4 of PC:PE:cholesterol:sulfatides. PtdIns(4,5)P_2_ liposomes were prepared by percent ratio of 50∶20∶10∶10∶10 of PC:PE:PS:cholesterol:PtdIns(4,5)P_2_. Controls were prepared by adjusting the ratios with PC. Lipid films were generated by lyophilization and hydrated in 20 mM Tris-HCl (pH 6.8) and 100 mM NaCl to 1 mg/mL at 60°C for 1 h. Liposomes were sonicated, pelleted, and suspended at 2.5 mg/mL in the same buffer. Ten µg of protein was incubated with 125 µg of total lipid for 20 min at 23°C. Liposome-bound and free-protein samples were separated by centrifugation and analyzed by SDS-PAGE.

### Surface Plasmon Resonance

SPR binding experiments were performed on a BIAcore X instrument using L1 sensorchips coated with ∼0.5 mM of mixed sulfatides or PtdIns(4,5)P_2_ 100 nm size-calibrated liposomes. The Dab2 N-PTB binding experiments were performed in 10 mM Tris-HCl (pH 7.4) and 100 mM NaCl. This buffer was used during the equilibration, association, and dissociation phases. Proteins were added to this buffer at the indicated concentrations. Regeneration of the lipid bilayer after the dissociation phase was carried out using 20 mM NaOH. The data were globally fit using BIAevaluation 3.0.

### Thrombin-Limited Proteolysis

Sulfatide and PtdIns(4,5)P_2_ liposomes were incubated with 10 µg of protein for 20 min at 23°C, centrifuged, and the pellets suspended in 50 µl of 20 mM Tris-HCl (pH 6.8) and 100 mM NaCl. Thrombin (0.05 units/µg protein) was added to both supernatants and pellets and aliquots were taken at 0, 8, 12, 16, and 20 h after digestion. Reactions were stopped with Laemmli buffer and analyzed by SDS-PAGE.

### Immunofluorescence Analysis

Studies on human platelets were carried out with ethical approval from the Office of Research Compliance at Virginia Tech, with written informed consent obtained from all donors. Washed platelets from healthy human volunteers (6×10^6^ platelets) were incubated with 1.9 µM of untagged N-PTB constructs for 5 min at 23°C in the presence of 0.25 g/L fibrinogen. Activation was initiated by the addition of 10 µM TRAP. Reactions were incubated at 23°C unless otherwise indicated.

Platelets were fixed with 3.7% formaldehyde in phosphate buffered saline (PBS) for 30 min. Next, 20% goat serum was added to the fixed platelets and 60% of the total fixed reaction was cytospun onto a Shandon-coated cytoslide. Slides were then washed twice with PBS for 10 min. Platelets were permeabilized with 0.5% Triton X-100 in PBS for 10 min and then blocked with 20% goat serum in PBS containing 0.1% Triton X-100 for 1 h at room temperature. Platelets were washed three times with PBS with 0.1% Triton for 10 min and analyzed as described [25] using anti-Dab2 (anti-p96; BD Transduction), Cy-3 conjugated goat anti-mouse antibody (Sigma), and Alexa488 conjugated phalloidin (5 U/mL; Invitrogen). Platelets were observed on an Olympus IX71 microscope, using a 100x NA 1.4 UPIanSApo objective lens. Images were captured with a charge-coupled device camera (Photometrics CoolSNAP HQ2CCD) and analyzed using SoftWorx software (Applied Precision). Mean pixel intensity was determined for at least 450 platelets from at least four fields. For inhibition assays, platelets were incubated with 0.25 g/L fibrinogen with either 28 µM chlorpromazine (3 min), 4 µM cytochalasin D (5 min), 1.5 µM filipin (60 min), 5 mM MBCD (20 min) (Sigma) or vehicle. N-PTB, N-PTB^4M^, or N-PTB^D66E^ (1.9 µM) was then added and the reaction incubated for 4 min. TRAP (10 µM) was then added to initiate the reactions and samples were processed as described above.

### Platelet Adhesion Assay

Non-tissue culture plates were incubated with fibrinogen (20 µg/mL) for 2 h at 37°C, washed with PBS and blocked with 5% fatty acid-free BSA for 24 h at 4°C. After three washes, 2×10^7^ platelets were added together with RGDS peptide, N-PTB, N-PTB^4M^, or N-PTB^D66E^ (1 µM each) and TRAP (10 µM) for 20 min at 37°C. Bound platelets were quantified as described [26].

### Flow Cytometry Analysis

A mixture of 50 µM of protein or peptide, 1 µg/mL final FITC-labeled PAC-1 antibody (BD Transduction), 5 µl APC-CD42b (BD Transduction), and 10 µM TRAP were mixed and incubated with 1×10^6^ washed human platelets for 10 min at 23°C. Platelets were fixed with 1% formalin for 15 min at 23°C. Bound PAC-1 antibody was measured using a FacsAria flow cytometer. For the expression of the endogenous integrin receptor assay, platelets were activated with 30 µM ADP and further incubated with liposomes (50 µg/mL) and in the absence or presence of Dab2 N-PTB constructs (1 µM). Platelets were processed as described above.

### Statistical Analysis

Statistics were analyzed using the Student's *t* test (p-value<0.05, statistically significant).

## Results and Discussion

### Identification of Sulfatide-Binding Sites in Dab2

**Figure 1:**
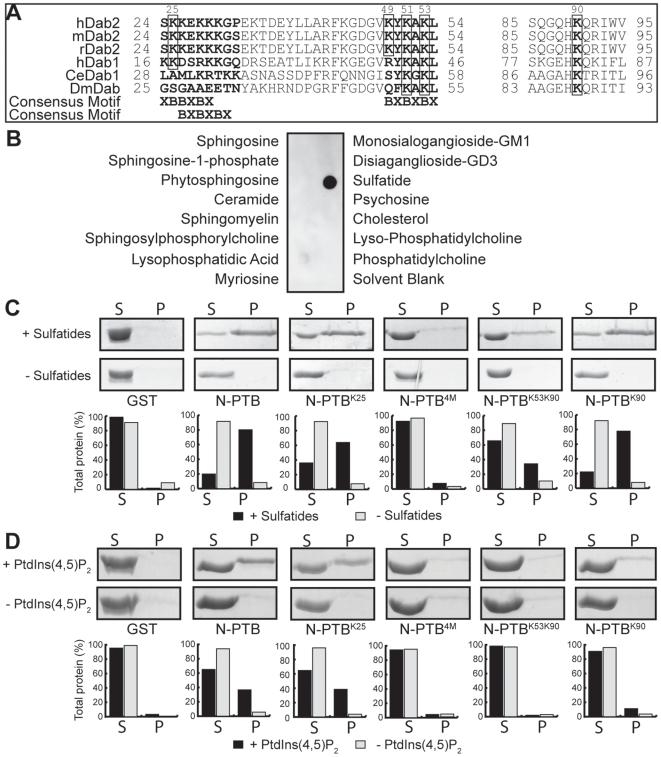
Dab2 N-PTB interacts with sulfatides. (A) Sequence alignment of Dab2 regions involved in sulfatide binding. Residues predicted to interact with sulfatides and PtdIns(4,5)P_2_ are boxed. Consensus motifs are indicated at the bottom. hDab2, *H. sapiens* Dab2; mDab2, *M. musculus* Dab2; rDab2, *R. norvegicus* Dab2; hDab1, *H. sapiens* Dab1; CeDab1, *C. elegans* Dab1; DmDab, *D. melanogaster* Dab. (B) Sphingolipid strips spotted with the indicated lipids were probed with 0.2 µg/ml of Glutathione S-transferase (GST)-N-PTB. (C) Liposome binding assay for N-PTB and its mutants in the presence (+) and absence (−) of sulfatides. S: supernatant; P: pellet. Bands were quantified using AlphaImager and normalized to the input (bars). The figure shows data from a single experiment that was repeated three times with similar results. (D) Same as C but in the presence and absence of PtdIns(4,5)P_2_. N-PTB, N-terminal Dab2 (amino acids 1-241); N-PTB^K25^, N-PTB Lys25Ala; N-PTB^4M^, N-PTB Lys25Ala, Lys49Ala, Lys51Ala and Lys53Ala; N-PTB^K53K90^, N-PTB Lys53Ala and Lys90Ala; N-PTB^K90^, N-PTB Lys90Ala.

Analysis of the amino acid sequence of the N-terminal region of Dab2, including its PTB domain, showed the presence of conserved positively charged residues that closely resemble two characteristic sulfatide-binding sites, the XBBXBX and BXBXBX motifs [27] (B, basic; X, any residue; Fig. 1A) described in other haemostatic proteins, including laminin-1 [28] and vWF [29]. Therefore, we analyzed the N-terminal region of Dab2 (residues 1-241; N-PTB) for binding to sulfatides in comparison with other sphingolipids found at the plasma membrane. Our results show that N-PTB specifically binds to sulfatides as analyzed by the protein-lipid overlay assay (Fig. 1B and Fig. S1A). Because the Dab2 PTB targets membranes [9], we then employed liposomes enriched with sulfatides to further investigate the interaction. We found that N-PTB binds to sulfatide liposomes (Fig. 1C and Fig. S1B). Single mutations to Ala designed within the putative sulfatide-binding sites (Lys25, Lys49, or Lys53) or a combination of two mutations (Lys25 and Lys49) reduced but did not eliminate sulfatide binding (Fig. 1C and Fig. S1C). However, a substitution of four positively charged residues (Lys25, Lys49, Lys51, and Lys53; N-PTB^4M^) to Ala (Fig. 1A) completely abolished sulfatide binding (Fig. 1C, Fig. S1A and B). Circular dichroism spectra of N-PTB^4M^ and mutants exhibit similar overall secondary structure content compared with N-PTB, indicating that mutations do not significantly perturb the global fold of the protein (Text S1, Fig. S2 and Table S1). Thus, these results identify two positively charged motifs in N-PTB that play a critical role in sulfatide recognition.

### Sulfatide- and PtdIns(4,5)P_2_-Binding Sites Share Lys53 in N-PTB

**Figure 2:**
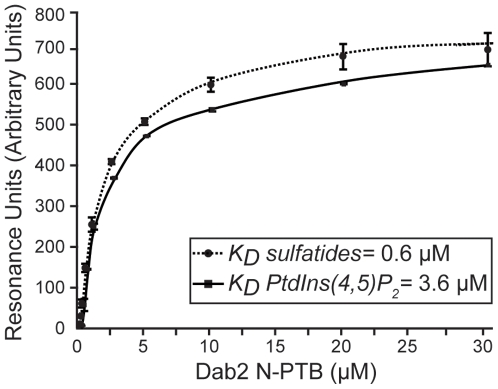
Kinetic analysis of lipid binding by Dab2 N-PTB. Interactions of N-PTB with immobilized sulfatide- (circles) and PtdIns(4,5)P_2_-liposomes (squares) were analyzed by SPR. Resonance units quantify the bound protein fraction at increasing protein concentrations. Data represent the average of two independent experiments.

The Dab2 PTB domain is known to bind phosphoinositides with a preference for PtdIns(4,5)P_2_ [16], independent of the receptor interaction site [30]. Two basic residues (Lys53 and Lys90) have been reported to play a critical role in PtdIns(4,5)P_2_ recognition [31]. Because we found that Lys53 is involved in sulfatide binding, we then examined whether Lys90 could also be critical for sulfatide recognition. Mutations in both Lys53 and Lys90 to Ala (N-PTB^K53K90^) reduced sulfatide binding by about 3-fold (Fig. 1C), similarly to a sole mutation at Lys53 (Fig. S1C). However, a single mutation at Lys90 (N-PTB^K90^) resembled sulfatide binding by N-PTB (Fig. 1C), indicating that Lys90 is not required for sulfatide recognition. As expected, N-PTB bound to PtdIns(4,5)P_2_ liposomes, whereas N-PTB^K53K90^ and N-PTB^4M^ did not (Fig. 1D). Unlike the Lys53 mutant, single or double mutations in the sulfatide-binding sites in N-PTB did not significantly affect PtdIns(4,5)P_2_ binding (Fig. 1D and Fig. S3). Mutations in Lys53 and Lys90 did not alter the overall secondary structure of the protein, indicating that they specifically abolished lipid binding (Text S1, Fig. S2 and Table S1). Although sulfatides and PtdIns(4,5)P_2_ require Lys53 for N-PTB recognition, it is unlikely that they can physiologically compete with each other for binding to Dab2 since sulfatides are present on external membrane surfaces [20], whereas PtdIns(4,5)P_2_ is found on the inner leaflet [32]. Next, we investigated the kinetics of lipid interaction with N-PTB by SPR. The N-PTB displayed reversible binding to either immobilized sulfatide or PtdIns(4,5)P_2_ liposomes, and both fit a two-state reaction with conformational change interaction model. The N-PTB bound to sulfatides with an estimated *K_d_* of 0.6 µM (χ2 = 2.6) (Fig. 2), a value similar to that reported for sulfatide binding by annexin V [33]. The N-PTB bound to PtdIns(4,5)P_2_ with a calculated *K_d_* of 3.6 µM (χ2 = 5.1) (Fig. 2), which is in the range of that reported for the Dab1 PTB domain [34].

### Sulfatides Protect N-PTB from Thrombin Cleavage

**Figure 3:**
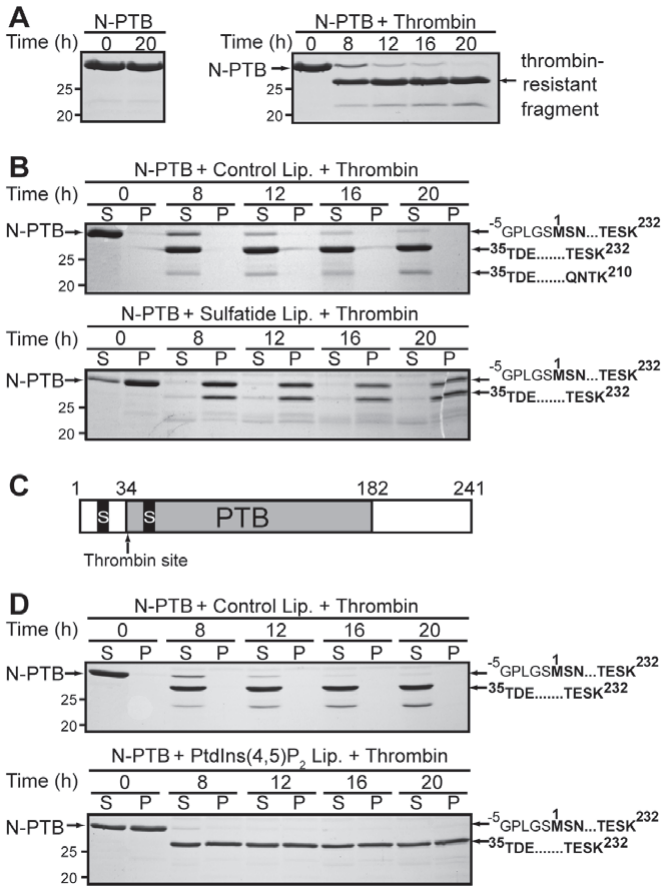
Sulfatides protect Dab2 N-PTB from thrombin proteolysis. (A) N-PTB was incubated in the absence (left) and presence (right) of thrombin and samples collected at the indicated times were analyzed by SDS-PAGE. (B) N-PTB was pre-incubated with control (top) and sulfatide liposomes (bottom) and incubated with thrombin. Boundaries of the amino acid sequence of trypsin-digested bands, obtained by mass spectrometry analysis, are indicated on the right. The sequence GPLGS is translated from the vector. (C) Diagram of the N-PTB region indicating sulfatide-binding (S) and thrombin cleavage sites. (D) N-PTB was pre-incubated with control (top) and PtdIns(4,5)P_2_ liposomes (bottom) and processed as described in A and B. The figure shows data from a single experiment that was repeated two times with similar results.

Platelets not only secrete molecules that facilitate blood coagulation but also participate in haemostasis by secreting various anti-coagulant proteins from their α-granules, thus, limiting the progression of blood clotting [2]. For example, during platelet activation, Dab2 is released from α-granules to the platelet surface and binds to the extracellular region of the αIIbβ3 integrin, where it exerts its anti-platelet aggregation activity [9]. Thrombin, a strong platelet agonist, cleaves Dab2 in its PTB domain; thus, Dab2 could be proteolyzed during thrombin-mediated platelet activation [9]. Our results indicate that thrombin cleaved N-PTB, leaving a protease-resistant product (Fig. 3A). To examine the role of Dab2-sulfatide binding in the context of thrombin-mediated platelet activation, N-PTB was pre-incubated with sulfatide liposomes and protein-lipid complexes were treated with thrombin. Interestingly, sulfatide liposomes partially protected N-PTB from thrombin cleavage (Fig. 3B). Mass spectrometry analyses of trypsin-digested, thrombin-resistant polypeptides indicated that the first sulfatide-binding site of N-PTB is located upstream of a putative thrombin cleavage site (Lys30, Gly31) and protected from cleavage when bound to sulfatide liposomes (Fig. 1A, 3B, and C). Although thrombin cleavage of Dab2 might lead to the loss of Dab2 inhibitory activity in a platelet aggregation assay [9], we hypothesize that Dab2 binding to sulfatides at the platelet surface could protect the protein from thrombin cleavage (our results) while contributing to blocking sulfatides needed for pro-coagulant interactions [20]. In contrast, PtdIns(4,5)P_2_ was unable to protect N-PTB from thrombin cleavage (Fig. 3D), indicating that the phosphoinositide does not have a direct role in Dab2 stabilization during thrombin-mediated platelet activation.

### Sulfatides Mediate Localization and Internalization of N-PTB

**Figure 4:**
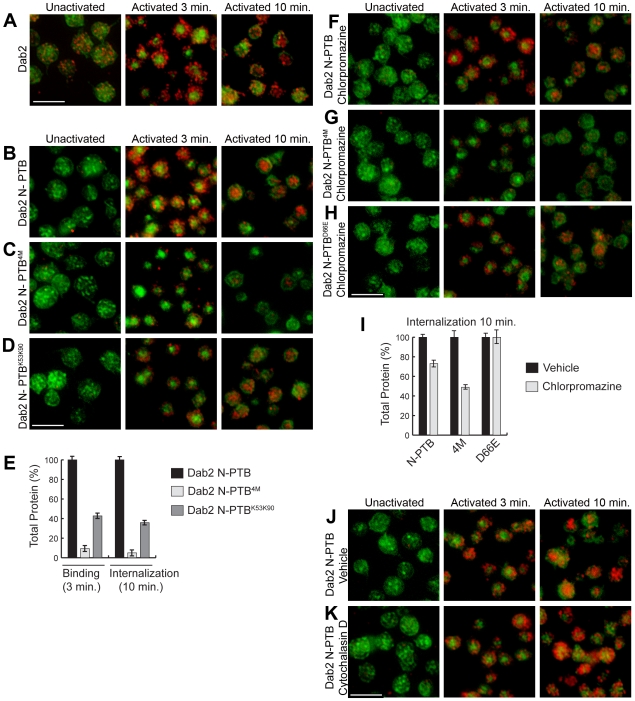
Role of sulfatides and PtdIns(4,5)P_2_ in Dab2 N-PTB localization. (A) Endogenous Dab2 (red) from washed platelets was monitored prior to activation (Unactivated), and 3 and 10 min after TRAP addition. N-PTB (B), N-PTB^4M^ (C), or N-PTB^K53K90^ (D) (all in red) were monitored at the same times. Phalloidin (green) stains actin stress fibers. (E) Quantification of binding (3 min) and internalization (10 min) of N-PTB, N-PTB^4M^, and N-PTB^K53K90^ are represented by bars. The average mean pixel intensity of approximately 450 platelets was quantified for each analysis. The average mean pixel intensity of the endogenous protein in a given experiment was then subtracted from the average mean pixel intensity of a sample containing exogenous protein to yield only the net mean pixel intensity generated by the exogenous proteins. The net mean pixel intensity values of the N-PTB mutant proteins were compared to the net mean pixel intensity of the wild type N-PTB (referred as 100%) at 3 min. The percentage of the protein that was internalized (10 minutes) for each construct was determined by multiplying the percentage of protein internalized at 10 minutes (compared to the wild type N-PTB at 10 minutes) by the amount of protein bound for each construct at 3 minutes. (F–H) Washed platelets were treated with chlorpromazine, further incubated with N-PTB (F), N-PTB^4M^ (G), or N-PTB^D66E^ (H) and processed as above. (I) Quantification of internalized (10 min) N-PTB, N-PTB^4M^, and N-PTB^D66E^ in the presence of chlorpromazine compared to their vehicle. Quantifications were performed as described in (E). (J–K) Washed platelets treated with vehicle (J) or cytochalasin D (K) and further incubated with N-PTB were processed as in A. In panels B–D, F–H and J–K, each experiment was normalized to that shown in A. The figure shows data from a single experiment that was repeated two times with similar results. N-PTB^D66E^, N-PTB Asp66Glu. Bar, 5 µm.

The N-PTB has been shown to bind to the integrin receptor [9] and sulfatides (this study). Thus, we hypothesize that two pools of Dab2 co-exist at the activated platelet surface. Consequently, we analyzed the subcellular localization of endogenous Dab2, as well as exogenously added N-PTB and N-PTB^4M^ during thrombin receptor-activating peptide (TRAP)-mediated platelet activation (Fig. 4 and Fig. S4A). Upon TRAP addition, endogenous Dab2 localized peripherally (Fig. 4A, Activated 3 min). Accordingly, the localization of N-PTB was indistinguishable from endogenous Dab2 (Fig. 4B, Activated 3 min). However, mutations in both sulfatide-binding motifs (N-PTB^4M^) reduced the localization of the protein at the activated platelet surface by about 90% (Fig. 4C, Activated 3 min), indicating a role for sulfatides in the localization of N-PTB. Thus, we propose that the remaining N-PTB^4M^ localized at the platelet surface (Fig. 4C, Activated 3 min) is most likely bound to the integrin receptor through its RGD (Arg64, Gly65, Asp66) motif, a sequence that specifically binds to integrin receptors [35]. This model is supported by findings that show Dab2 PTB binds to the αIIb domain of the integrin receptor through the Dab2 RGD motif [9]. Indeed, mutation of Asp66 to Glu (D66E) abolishes Dab2 interaction with the receptor [9]. Our results show that N-PTB^D66E^ binds to a lesser extent to the activated platelet surface (Fig. S4B and C). To confirm our model, we investigated the direct binding of the Dab2 N-PTB constructs with the endogenous integrin αIIbβ3 receptor from activated platelets by pull-down and immunoblotting. As expected, both Dab2 N-PTB and N-PTB^4M^ bound to the receptor, whereas N-PTB D66E did not (Text S1 and Fig. S4D). In addition, a construct containing mutations in both sulfatide and the integrin receptor binding sites (Dab2 N-PTB^5M^; K25A/K49A/K51A/K53A/D66E) was unable to associate with the integrin receptor, mimicking the effect of the single D66E mutation in N-PTB (Fig. S4D). Since this mutation does not abrogate binding to sulfatides, but sulfatide-binding mutations in N-PTB^4M^ and N-PTB^5M^ do (Fig. 1C and S4E), our data support a model in which Dab2 N-PTB^D66E^ is preferentially available for sulfatide binding at the platelet surface.

Interestingly, when platelets were fixed 10 min after activation, endogenous Dab2, as well as N-PTB, N-PTB^4M^, and N-PTB^D66E^ exhibited complete internalization (Fig. 4A–C, E and Fig. S4B, Activated 10 min). However, mutants showed less total internalized protein when compared with the wild-type form (Fig. 4B, C, and Fig. S4B Activated 10 min; E, Internalization 10 min), as expected since the amount of protein bound to the surface is also comparably reduced (Fig. 4B, C, Activated 3 min; E, Binding 3 min). Since platelets are known to internalize the integrin receptor [36], the accumulation of intracellular protein over time is anticipated, especially for those mutant proteins (*i.e.*, N-PTB^4M^) that favor integrin binding (Fig. 4E). Mutation in the PtdIns(4,5)P_2_-binding site, N-PTB^K53K90^, decreased the membrane localization of the protein by 58% (Fig. 4D, Activated 3 min; E, Binding 3 min), indicating that Lys53 is critical for both sulfatide binding and localization of Dab2 at the platelet surface. Furthermore, 10 min post-activation, the surface-bound N-PTB^K53K90^ was internalized (Fig. 4D, Activated 10 min; E, Internalization 10 min). This result indicates that PtdIns(4,5)P_2_ binding is not required for internalization of the extracellular N-PTB and that N-PTB^K53K90^, with reduced sulfatide-binding properties (Fig. 1C), is likely internalized in an integrin receptor-dependent manner. PtdIns(4,5)P_2_, however, could interact with cytosolic Dab2 since this phosphoinositide mediates Dab2 recruitment, local membrane destabilization, and deformation during endocytosis [16].

### N-PTB Is Internalized by an Actin Cytoskeleton-Dependent Mechanism

To determine where Dab2 is delivered upon its internalization later in platelet activation, we carried out immunofluorescence and co-localization analyses with platelet factor 4 (PF4), a marker of α-granules (Text S1 and Fig. S5). Interestingly, we found that Dab2 co-localizes with PF4 before and after degranulation, suggesting that the internalized protein is recycled and again stored in α-granules. This is in agreement with the proposed mechanism of constitutive trafficking of internalized proteins to α-granules throughout the lifespan of the platelet [2]. To further investigate the mechanism by which N-PTB is internalized in platelets, we employed inhibitors of actin polymerization and endocytosis. Platelets exhibited peripheral N-PTB shortly after their activation in the presence of all inhibitors tested (Fig. 4F–H, J, K Activated 3 min and Fig. S6). No inhibition of N-PTB internalization was observed when activated platelets were incubated with either methyl-β-cyclodextrin (MBCD), a cholesterol-depleting agent for caveolae-mediated endocytosis [37], or filipin, an inhibitor of caveolae formation [38] (Fig. S6A and B, Activated 10 min). Intriguingly, chlorpromazine, an inhibitor of clathrin-mediated endocytosis [39], weakly inhibited N-PTB internalization (Fig. 4F, I). When chlorpromazine was tested in the presence of N-PTB^4M^, internalization of the protein was reduced by more than 50%, whereas N-PTB^D66E^ was unaffected (Fig. 4G and H, Activated 10 min; I, Fig. S6C–E). Since integrin receptors are internalized by clathrin-mediated endocytosis [40], we propose that chlorpromazine preferentially inhibits the internalization of the integrin receptor-bound pool of N-PTB. Finally, internalization of N-PTB was completely inhibited in the presence of cytochalasin D, an actin polymerization inhibitor [41] (Fig. 4J and K, Activated 10 min). Overall, our results support a mechanism of sulfatide-mediated Dab2 internalization that is lipid raft and clathrin-independent but actin cytoskeleton-dependent.

### Sulfatides Modulate the Availability of Dab2 for the Integrin Receptor

**Figure 5:**
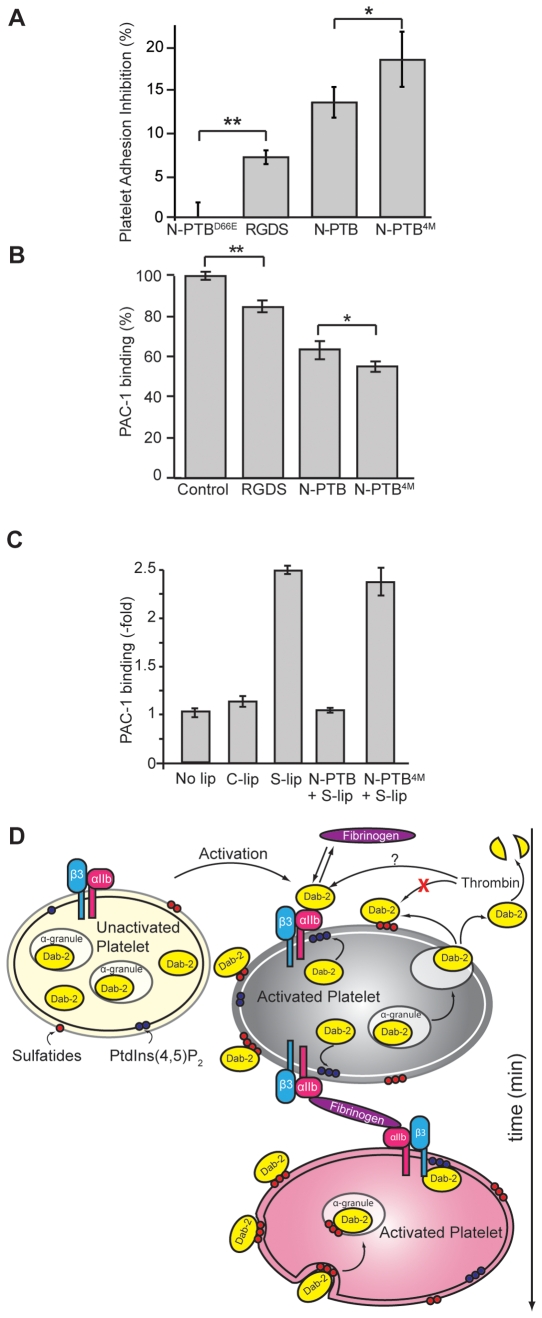
Two pools of Dab2 exist at the activated platelet surface. (A) Washed platelets were activated in fibrinogen-coated wells in the presence of N-PTB^D66E^, RGDS peptide, N-PTB, or N-PTB^4M^. Inhibition of platelet adhesion was quantified compared to N-PTB^D66E^. Data represent the results of two independent experiments. *, *P*<0.03; **, *P*<0.01. (B) Washed platelets were incubated with PAC-1 antibody in the presence of control peptide (PPPVKKRAAKCLLL), RGDS, N-PTB, or N-PTB^4M^ and binding to the integrin receptor monitored by flow cytometry. Data represent the results of three independent experiments. *, *P*<0.03; **, *P*<0.002. (C) The Dab2 N-PTB sequesters sulfatides needed for integrin receptor expression at the activated platelet surface. Activated platelets (No lip) were incubated with liposomes without (C-lip) or with sulfatides (S-lip), and in the absence or presence of either N-PTB (N-PTB+S-lip) or N-PTB^4M^ (N-PTB^4M^+S-lip). Expression of the integrin receptor was quantified using a PAC-1 antibody. Data represent the results of two independent experiments. (D) Proposed model for the role of sulfatides in partitioning Dab2 during platelet activation. Upon platelet activation and degranulation, Dab2 is released extracellularly and is partitioned between the integrin receptor (to inhibit platelet aggregation) and the membrane surface by interaction with sulfatides (red balls). Dab2 surface binding is transient since the protein is internalized shortly by an actin-dependent mechanism and accumulated intracellularly in α-granules. Binding of cytoplasmic Dab2 to PtdIns(4,5)P_2_ (blue balls) may contribute to anchoring the protein to the cytosolic side of the platelet membrane.

Binding of fibrinogen to platelets mediates aggregation during blood clotting [42], an event regulated by Dab2 PTB, which competes with fibrinogen for integrin binding at the platelet surface [9]. To understand whether sulfatide binding by N-PTB governs integrin receptor function in platelets, we performed an adhesion assay. As expected, N-PTB^D66E^ did not affect platelet adhesion to fibrinogen, whereas RGDS, a low-affinity fibrinogen receptor antagonist [43], and N-PTB reduced platelet adhesion by about 7 and 13.5%, respectively (Fig. 5A). In agreement with our model, N-PTB^4M^ inhibited cell adhesion more efficiently (18.5%) (Fig. 5A), suggesting that abolishing sulfatide binding makes a larger pool of N-PTB available to compete with fibrinogen for binding to the integrin receptor. In support of this hypothesis, N-PTB reduced PAC-1 antibody binding to the integrin receptor by 36%, whereas N-PTB^4M^ decreased it by about 45% when analyzed by flow cytometry (Fig. 5B). We further investigated the role of sulfatides in the integrin receptor and Dab2 functions in activated platelets (Fig. 5C). Activated platelets increased the levels of the integrin receptor at their surface in the presence of sulfatide-enriched liposomes, consistent with the hypothesis that sulfatides activate the integrin receptor via signaling through P-selectin [44]. In the presence of Dab2 N-PTB, however, the expression levels of the integrin receptor mirrored the levels observed in the absence of exogenous sulfatides (Fig. 5C), indicating that N-PTB, but not N-PTB^4M^, sequestered sulfatide-liposomes from the platelet surface.

### Proposed Model for Lipid-Mediated Dab2 Action

Based on our results, we propose the existence of two pools of Dab2 at the surface of activated platelets (Fig. 5D). One pool negatively regulates platelet aggregation by competing with fibrinogen for binding to the αIIbβ3 integrin receptor, whereas a second pool of Dab2 binds to sulfatides. Thrombin cleaves the nonsulfatide-bound pool of Dab2 and, thus, controls the extent of clotting. Instead, the sulfatide-bound pool remains protected from thrombin cleavage and modulates the clotting response by sequestering Dab2 away from binding to the αIIbβ3 integrin receptor. Interestingly, whereas the integrin receptor pool appears to be specifically internalized by clathrin-mediated endocytosis, internalization of both Dab2 pools depends upon the dynamic nature of the actin cytoskeleton.

Sulfatides are present at the surface of blood cells including myeloid cells, erythrocytes, and platelets [45], [46], [47]. Hence, our findings might extend to other mediators of blood clotting. For example, the haemostatic glycoprotein vWF also binds both sulfatides and the αIIbβ3 integrin receptor, through its RGD motif, at the platelet surface [48]. Moreover, activated platelets express P-selectin, a transmembrane protein known to bind sulfatides and necessary to stabilize platelet and heterotypic aggregates [22], [44]. The major questions to be addressed are what mechanism dominates and under what circumstances, and how can these mechanisms be triggered in the context of our model.

Upon platelet activation, integrin receptors mediate clustering of PtdIns(4,5)P_2_ at the inner leaflet of the plasma membrane [49]. Activated platelets recycle integrin receptor, thus, downregulating the adhesiveness of platelets later in aggregation [36]. Since cytosolic Dab2 has been shown to bind to the β3 subunit of the integrin receptor [50], we speculate that PtdIns(4,5)P_2_ docks cytoplasmic Dab2 at the platelet membrane facilitating its endocytic function.

Cytosolic Dab1 is expressed exclusively in the brain where it participates in the Reelin signaling pathway and is required for the regulation of neuronal migration and positioning during embryonic development [51]. Sulfatides are the major constituent of the myelin sheath in the central and peripheral nervous system and they are found at the extracellular leaflet of the membrane of oligodendrocytes and Schwann cells [52]. In astrocytes and neurons, however, sulfatides are located intracellularly in large vesicles [53], [54]. Since human Dab1 exhibits canonical sulfatide-binding motifs at the N-terminus (Fig. 1A), it will be of interest to study whether the function of this protein is regulated by intracellular sulfatides in neuronal cells.

In conclusion, our work is the first demonstration that the protein Dab2, a platelet aggregation inhibitor, specifically binds to sulfatides and that this interaction modulates the progression of platelet activation. These findings further contribute to our understanding of how the dynamics of the ligand-dependent partitioning of Dab2 modulates platelet aggregation, thus, promoting the development of novel antiplatelet strategies.

## Supporting Information

Text S1 Supplementary text file.
(0.05 MB DOC)

Table S1 Prediction of secondary structure composition of Dab2 N-PTB and its mutants. All secondary structural elements content are in %. R and D represent predicted regular and distorted secondary structure elements, respectively. NRMSD is the normalized root mean square difference of the experimental and calculated spectra. Predictions were generated using DICHROWEB and deconvoluted using CDSSTR.
(0.53 MB TIF)

Figure S1 Identification of the residues of Dab2 N-PTB involved in sulfatide binding. (A) Membranes spotted with the indicated amount of sulfatides were probed with 1 µg/ml GST or GST-Dab2 N-PTB constructs. (B) Dose-dependent binding of the Dab2 N-PTB and Dab2 PTB4M was tested using sulfatide-enriched liposomes. (C) Liposome binding assay of the indicated Dab2 N-PTB mutants were assayed in the presence (+) and absence (−) of sulfatides. ‘S’ and ‘P’ represent proteins present in supernatant and pellet fractions, respectively. Bands were quantified using AlphaImager and normalized to the input amount. The figure shows data from a single experiment that was repeated three times with similar results. N-PTBK49, N-PTB Lys49Ala; N-PTBK53, N-PTB Lys53Ala; N-PTBK25K49, N-PTB Lys25Ala and Lys49Ala.
(1.73 MB TIF)

Figure S2 Mutations in Dab2 N-PTB do not alter the overall secondary structure of the protein. Far-UV circular dichroism spectra of Dab2 N-PTB and its mutants (5 µM each) were converted to mean residue ellipticity using DICHROWEB and deconvoluted using CDSSTR.
(0.73 MB TIF)

Figure S3 Sulfatide-binding amino acids Lys25 and Lys49 are dispensable for PtdIns(4,5)P2 binding. Mutants N-PTBK49, N-PTBK53 and N-PTBK25K49 were analyzed for PtdIns(4,5)P2 binding by liposome-binding assay in the presence (+) and absence (−) of PtdIns(4,5)P2. ‘S’ and ‘P’ represent proteins present in supernatant and pellet fractions, respectively. Bands were quantified using AlphaImager and normalized to the input amount. The figure shows data from a single experiment that was repeated three times with similar results.
(0.84 MB TIF)

Figure S4 Mutation of Asp66 to Glu in Dab2 N-PTB reduces binding of the protein to the platelet surface but it does not affect sulfatide binding. (A) Washed human platelets were incubated with bovine serum albumin (BSA; 1.9 µM) for 5 min at room temperature. Samples were fixed prior to activation (Unactivated), 3 min (Activated 3 min), and 10 min (Activated 10 min), after the addition of 10 µM TRAP and localization was monitored by immunofluorescence. (B) Mutation of the RGD motif reduces Dab2 N-PTB (Dab2 N-PTBD66E) localization at the platelet surface. Washed human platelets were incubated with Dab2 N-PTBD66E and processed as described in A. (C) Quantification of binding (3 min) and internalization (10 min) of Dab2 N-PTB and N-PTBD66E are represented by diagram bars. Approximately 450 platelets were quantified for each analysis as described in Methods. The percentage of the protein that was internalized (10 min) for each construct was determined by multiplying the percentage of protein internalized at 10 min (compared to the wild type N-PTB at 10 min) by the amount of protein bound for each construct at 3 min. Scale bar: 5 µm. (D) Binding of Dab2 N-PTB constructs with the endogenous αIIbβ3 integrin receptor of activated platelets. The indicated constructs (as GST fusion proteins-bound beads) were incubated with activated platelets for 1 h at room temperature. After lysis, beads were washed, samples subjected by SDS-PAGE and analyzed by western blotting. Bands were quantified using using AlphaImager and normalized to the input amount. The figure shows data from a single experiment that was repeated two times with similar results. (E) Liposome binding assay of N-PTB, N-PTB4M, N-PTBD66E and N-PTB5M was carried out in the presence (+) and absence (−) of sulfatides. ‘S’ and ‘P’ represent proteins present in supernatant and pellet fractions, respectively. Bands were quantified using AlphaImager and normalized to the input amount. The figure shows data from a single experiment that was repeated two times with similar results.
(3.46 MB TIF)

Figure S5 Internalized Dab2 co-localizes with PF4 in activated platelets. Dab2 (red) intracellular localization in unactivated (A) and activated (B) platelets was compared with PF4 (green), a marker of α-granules (green). The merge reveals the co-localization of Dab2 with the α-granule compartments.
(0.16 MB TIF)

Figure S6 Analysis of platelet localization of the Dab2 N-PTB constructs using inhibitors of endocytosis. (A–B) Washed human platelets were pre-incubated with MBCD (A) or filipin (B) and further incubated with Dab2 N-PTB (1.9 µM) for 5 min at room temperature. Samples were fixed prior to activation (Unactivated), 3 min (Activated 3 min), and 10 min (Activated 10 min), after the addition of 10 µM TRAP and localization was monitored by immunofluorescence. (C–E) Vehicle controls for the chlorpromazine experiment shown in Figure 4F–H. Scale bar: 5 µm. The figure shows data from a single experiment that was repeated two times with similar results.
(1.18 MB TIF)
