# Supplementary material for: Sulfatides Partition Disabled-2 in Response to Platelet Activation
Source: PLoS One. 2009 Nov 24;4(11):e8007. doi: 10.1371/journal.pone.0008007 (PMC2778132; doi:10.1371/journal.pone.0008007)
Supplement: Text S1 — Supplementary text file. (0.05 MB DOC) [file pone.0008007.s001.doc]

**Supplemental Information**

*Sulfatides partition Disabled-2 in response to platelet activation*

Karen E. Drahos, John D. Welsh, Carla V. Finkielstein, and Daniel G. S. Capelluto

**Circular Dichroism Spectroscopy:** Spectra were collected using a Jasco J-720 spectropolarimeter. Far-UV circular dichroism spectra were measured for Dab2 N-PTB constructs (5μM in 5mM Tris-HCl, pH 6.8, 100 mM KF, 100 μM DTT) using a 1mm-slit-width cuvette. Five accumulated scans were recorded for each sample from 240 to 190 nm at 20°C using a bandwidth of 1-nm and a response time of 1 s at a scan speed of 20 nm/min. The background of the buffer alone was subtracted from each spectrum. Raw data were converted to mean residue ellipticity and analyzed for secondary structure composition using DICHROWEB [1] and deconvoluted using CDSSTR [2].

**Pull-down Assay:** A total of 15 μg of GST-Dab2 N-PTB-bound beads were incubated in Tyrode’s Albumin Buffer containing 10 μM TRAP. Reactions were initiated by the addition of 100 μL of washed platelets (6x10^8^ cells/mL) in Tyrode’s Albumin Buffer. Reactions were incubated for one hour at room temperature. The beads were pulled down by centrifugation, the pellet resuspended in 500 μL of pull-down buffer (20 mM Tris-HCl (pH 7.5), 100 mM NaCl, 5 mM EDTA, 0.1% Triton-X100) and incubated for 15 min at room temperature. Then, beads were washed twice with the same buffer, twice with the same buffer containing 1 M NaCl, resuspended and boiled in Laemmli sample buffer. Samples were subjected to SDS-PAGE and transferred to PVDF membranes. Membranes were blocked with 5% nonfat dry milk in 20 mM Tris-HCl (pH 7.5), 137 mM NaCl and 0.025% Tween-20 and probed with anti-CD41 (Immunotech, SZ-22 clone) or anti-p96 (BD Transduction) overnight at 4°C. Membranes were washed and incubated with anti-mouse-horseradish peroxidase conjugated antibody (GE Healthcare) for 2 h at room temperature. Detection was performed using the enhanced chemiluminiscence (ECL) reagent (Pierce).

**Immunofluorescence Analysis:** Washed platelets (6x10^6^ platelets) were incubated for 5 min at 23°C with 0.25 g/L fibrinogen. Activation was initiated by the addition of 10 μM TRAP. Reactions were incubated at 23°C unless otherwise indicated. Platelets were fixed with 3.7% formaldehyde in PBS for 30 min. Next, 20% goat serum was added to the fixed platelets and 60% of the total fixed reaction was cytospun onto a Shandon-coated cytoslide. Slides were then washed twice with PBS for 10 min. Platelets were permeabilized with 0.5% Triton X-100 in PBS for 10 min and then blocked with 20% goat serum in PBS containing 0.1% Triton X-100 for 1 h at room temperature. Platelets were washed three times with PBS with 0.1% Triton for 10 min and analyzed as described using anti-Dab2 (anti-p96; BD Transduction), anti-PF4 (Santa Cruz Biotechnologies), Cy-3 conjugated goat anti-mouse antibody (Sigma) and FITC-conjugated anti-goat antibody (Jackson Laboratories). Platelets were observed on an Olympus IX71 microscope, using a 100x NA 1.4 UPIanSApo objective lens. Images were captured with a charge-coupled device camera (Photometrics CoolSNAP HQ2CCD) and analyzed using SoftWorx software (Applied Precision).

1. Whitmore L, Wallace BA (2004) DICHROWEB, an online server for protein secondary structure analyses from circular dichroism spectroscopic data. Nucleic Acids Res 32: W668-673.

2. Sreerama N, Woody RW (2004) Computation and analysis of protein circular dichroism spectra. Methods Enzymol 383: 318-351.
