# Supplementary figures and images for: Sulfatides Partition Disabled-2 in Response to Platelet Activation
Source: PLoS One. 2009 Nov 24;4(11):e8007. doi: 10.1371/journal.pone.0008007 (PMC2778132; doi:10.1371/journal.pone.0008007)

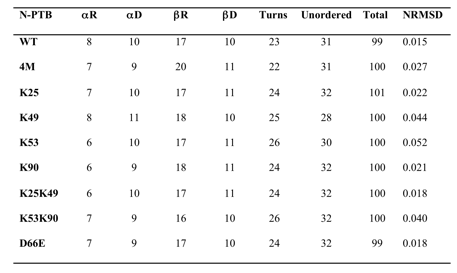

Supplement: Table S1 — Prediction of secondary structure composition of Dab2 N-PTB and its mutants. All secondary structural elements content are in %. R and D represent predicted regular and distorted secondary structure elements, respectively. NRMSD is the normalized root mean square difference of the experimental and calculated spectra. Predictions were generated using DICHROWEB and deconvoluted using CDSSTR. (0.53 MB TIF) [file pone.0008007.s002.tif]

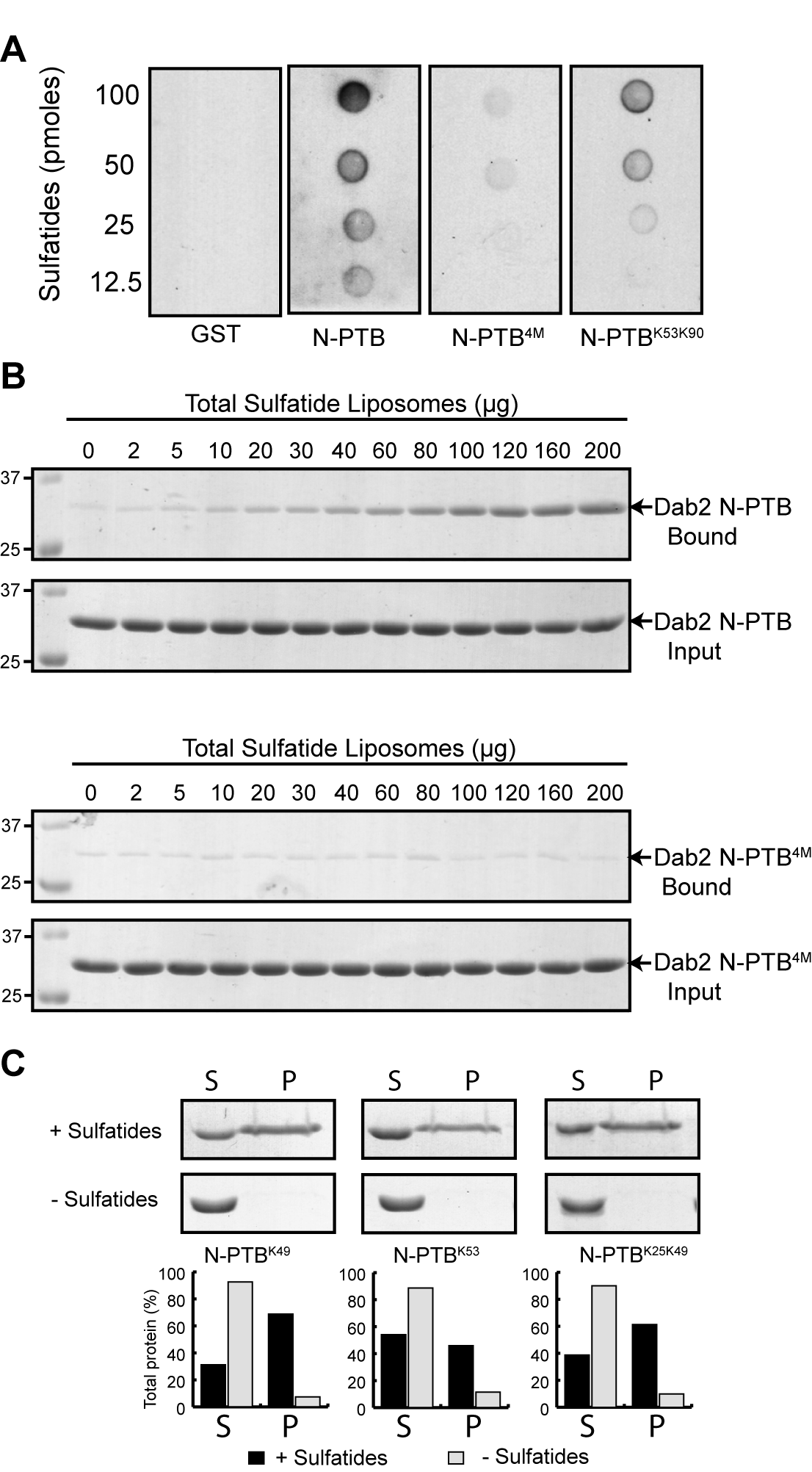

Supplement: Figure S1 — Identification of the residues of Dab2 N-PTB involved in sulfatide binding. (A) Membranes spotted with the indicated amount of sulfatides were probed with 1 µg/ml GST or GST-Dab2 N-PTB constructs. (B) Dose-dependent binding of the Dab2 N-PTB and Dab2 PTB4M was tested using sulfatide-enriched liposomes. (C) Liposome binding assay of the indicated Dab2 N-PTB mutants were assayed in the presence (+) and absence (−) of sulfatides. ‘S’ and ‘P’ represent proteins present in supernatant and pellet fractions, respectively. Bands were quantified using AlphaImager and normalized to the input amount. The figure shows data from a single experiment that was repeated three times with similar results. N-PTBK49, N-PTB Lys49Ala; N-PTBK53, N-PTB Lys53Ala; N-PTBK25K49, N-PTB Lys25Ala and Lys49Ala. (1.73 MB TIF) [file pone.0008007.s003.tif]

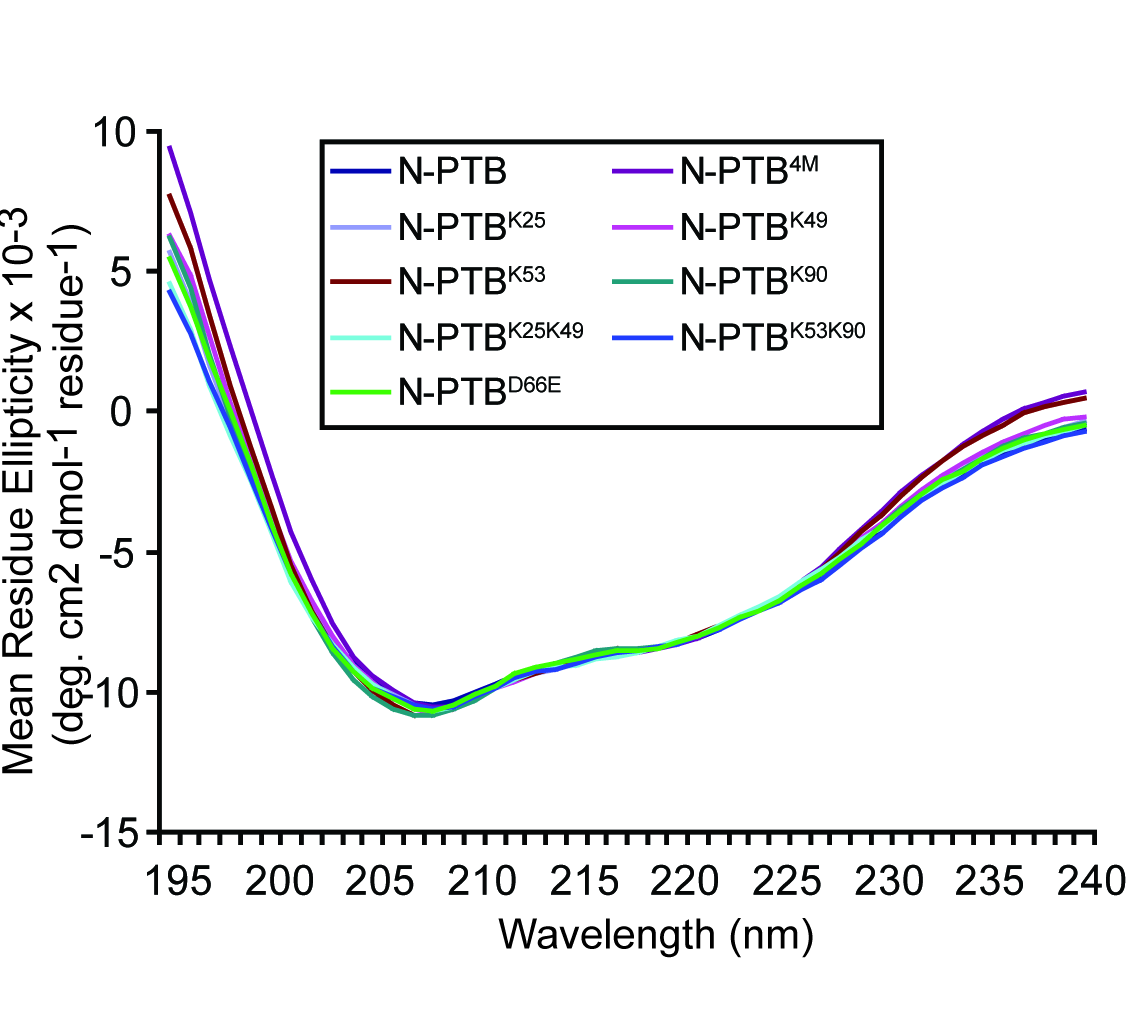

Supplement: Figure S2 — Mutations in Dab2 N-PTB do not alter the overall secondary structure of the protein. Far-UV circular dichroism spectra of Dab2 N-PTB and its mutants (5 µM each) were converted to mean residue ellipticity using DICHROWEB and deconvoluted using CDSSTR. (0.73 MB TIF) [file pone.0008007.s004.tif]

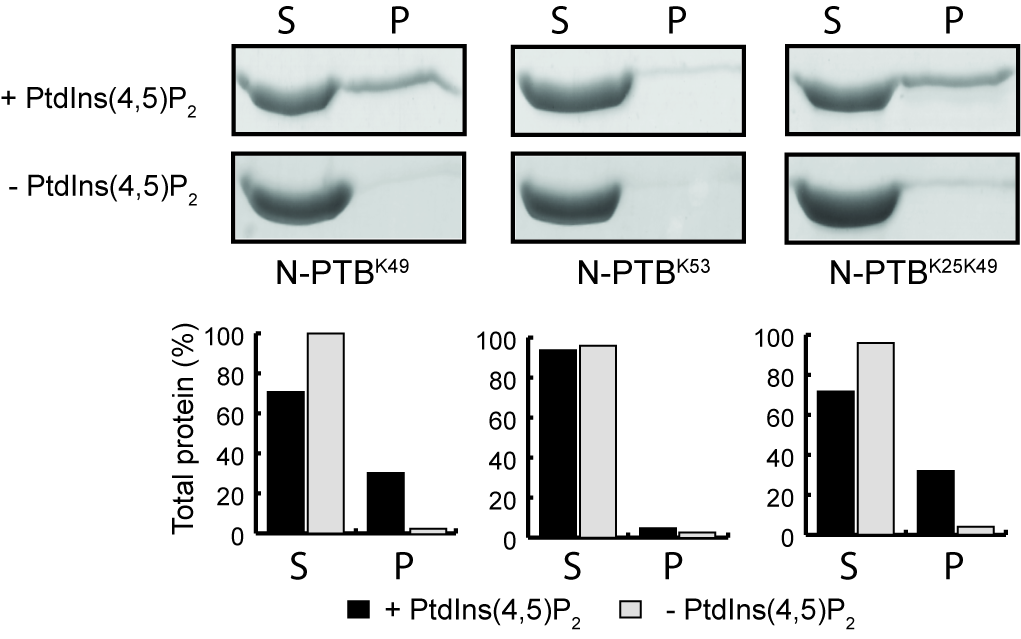

Supplement: Figure S3 — Sulfatide-binding amino acids Lys25 and Lys49 are dispensable for PtdIns(4,5)P2 binding. Mutants N-PTBK49, N-PTBK53 and N-PTBK25K49 were analyzed for PtdIns(4,5)P2 binding by liposome-binding assay in the presence (+) and absence (−) of PtdIns(4,5)P2. ‘S’ and ‘P’ represent proteins present in supernatant and pellet fractions, respectively. Bands were quantified using AlphaImager and normalized to the input amount. The figure shows data from a single experiment that was repeated three times with similar results. (0.84 MB TIF) [file pone.0008007.s005.tif]

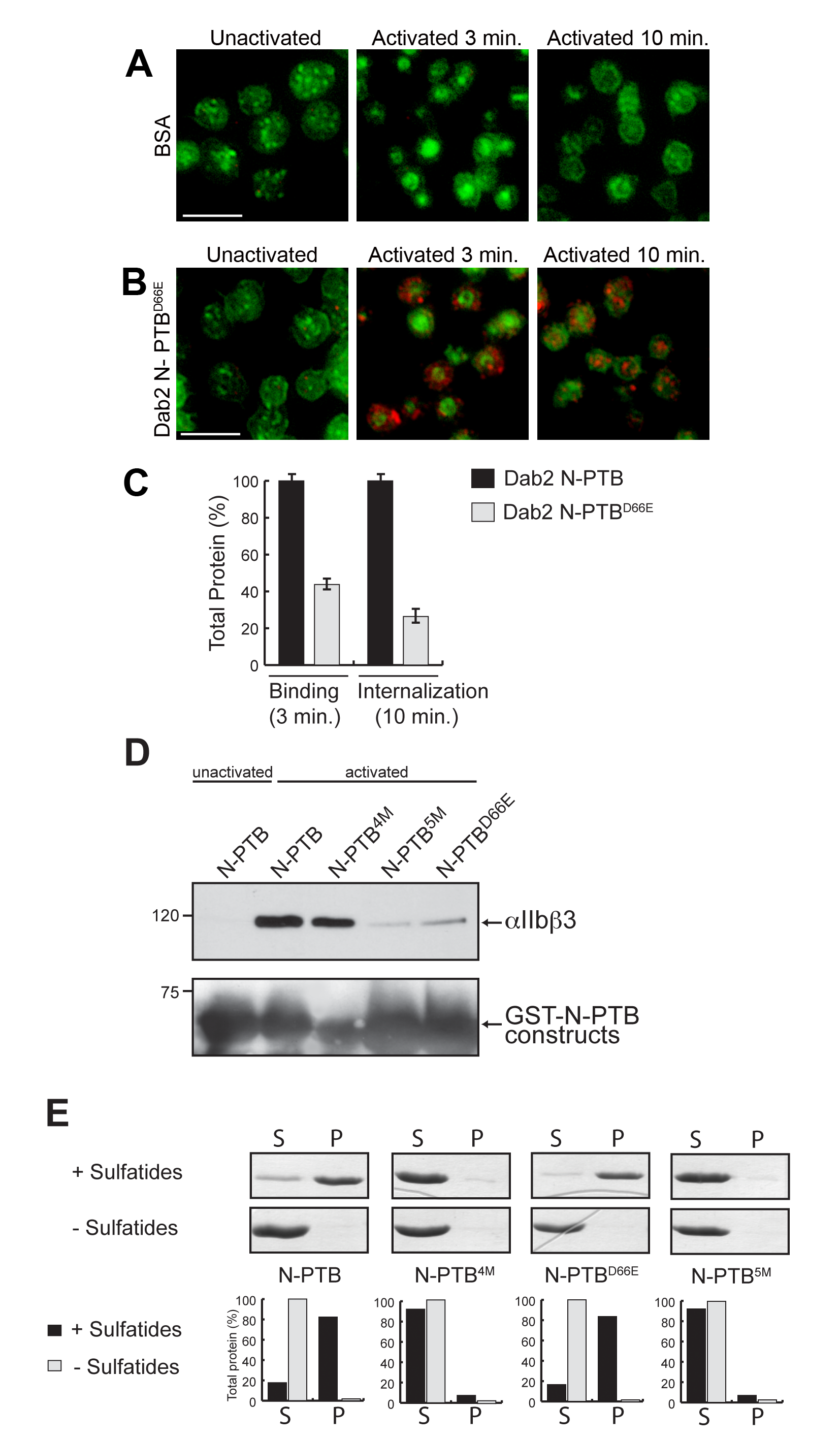

Supplement: Figure S4 — Mutation of Asp66 to Glu in Dab2 N-PTB reduces binding of the protein to the platelet surface but it does not affect sulfatide binding. (A) Washed human platelets were incubated with bovine serum albumin (BSA; 1.9 µM) for 5 min at room temperature. Samples were fixed prior to activation (Unactivated), 3 min (Activated 3 min), and 10 min (Activated 10 min), after the addition of 10 µM TRAP and localization was monitored by immunofluorescence. (B) Mutation of the RGD motif reduces Dab2 N-PTB (Dab2 N-PTBD66E) localization at the platelet surface. Washed human platelets were incubated with Dab2 N-PTBD66E and processed as described in A. (C) Quantification of binding (3 min) and internalization (10 min) of Dab2 N-PTB and N-PTBD66E are represented by diagram bars. Approximately 450 platelets were quantified for each analysis as described in Methods. The percentage of the protein that was internalized (10 min) for each construct was determined by multiplying the percentage of protein internalized at 10 min (compared to the wild type N-PTB at 10 min) by the amount of protein bound for each construct at 3 min. Scale bar: 5 µm. (D) Binding of Dab2 N-PTB constructs with the endogenous αIIbβ3 integrin receptor of activated platelets. The indicated constructs (as GST fusion proteins-bound beads) were incubated with activated platelets for 1 h at room temperature. After lysis, beads were washed, samples subjected by SDS-PAGE and analyzed by western blotting. Bands were quantified using using AlphaImager and normalized to the input amount. The figure shows data from a single experiment that was repeated two times with similar results. (E) Liposome binding assay of N-PTB, N-PTB4M, N-PTBD66E and N-PTB5M was carried out in the presence (+) and absence (−) of sulfatides. ‘S’ and ‘P’ represent proteins present in supernatant and pellet fractions, respectively. Bands were quantified using AlphaImager and normalized to the input amount. The figure shows data from a single experiment that [file pone.0008007.s006.tif]

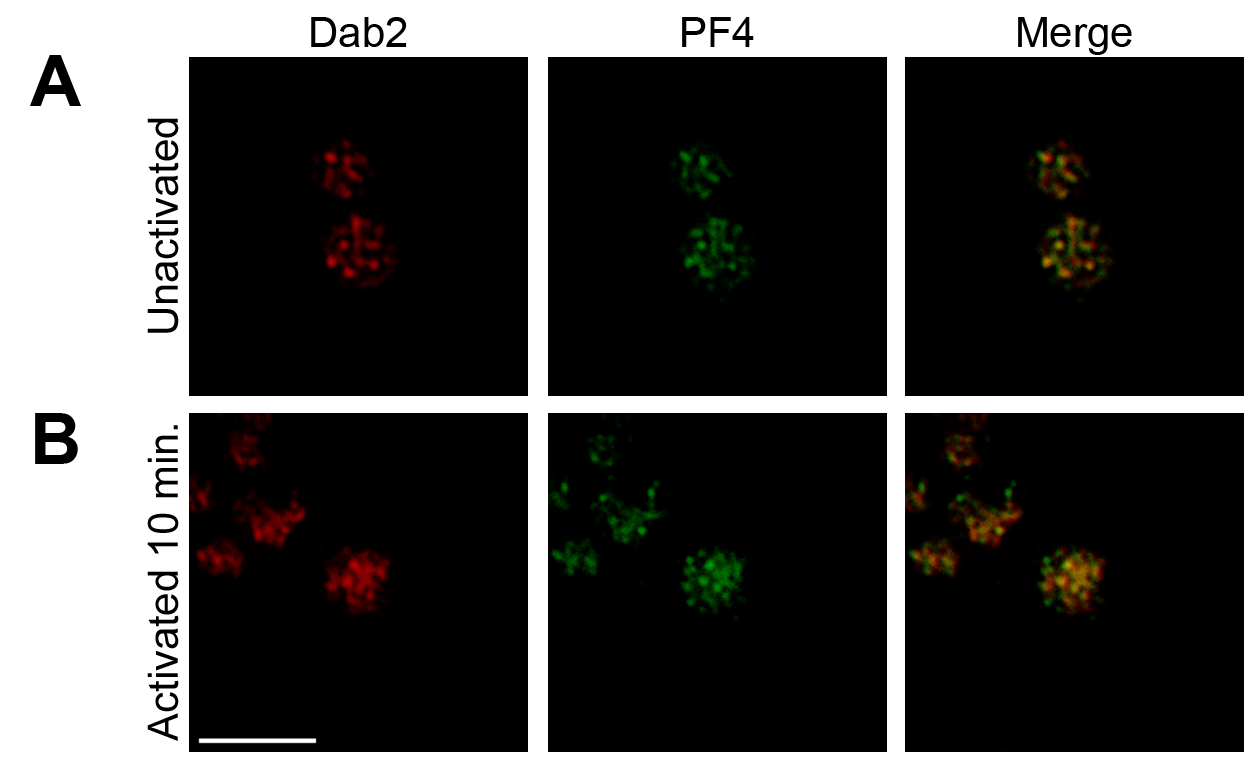

Supplement: Figure S5 — Internalized Dab2 co-localizes with PF4 in activated platelets. Dab2 (red) intracellular localization in unactivated (A) and activated (B) platelets was compared with PF4 (green), a marker of α-granules (green). The merge reveals the co-localization of Dab2 with the α-granule compartments. (0.16 MB TIF) [file pone.0008007.s007.tif]

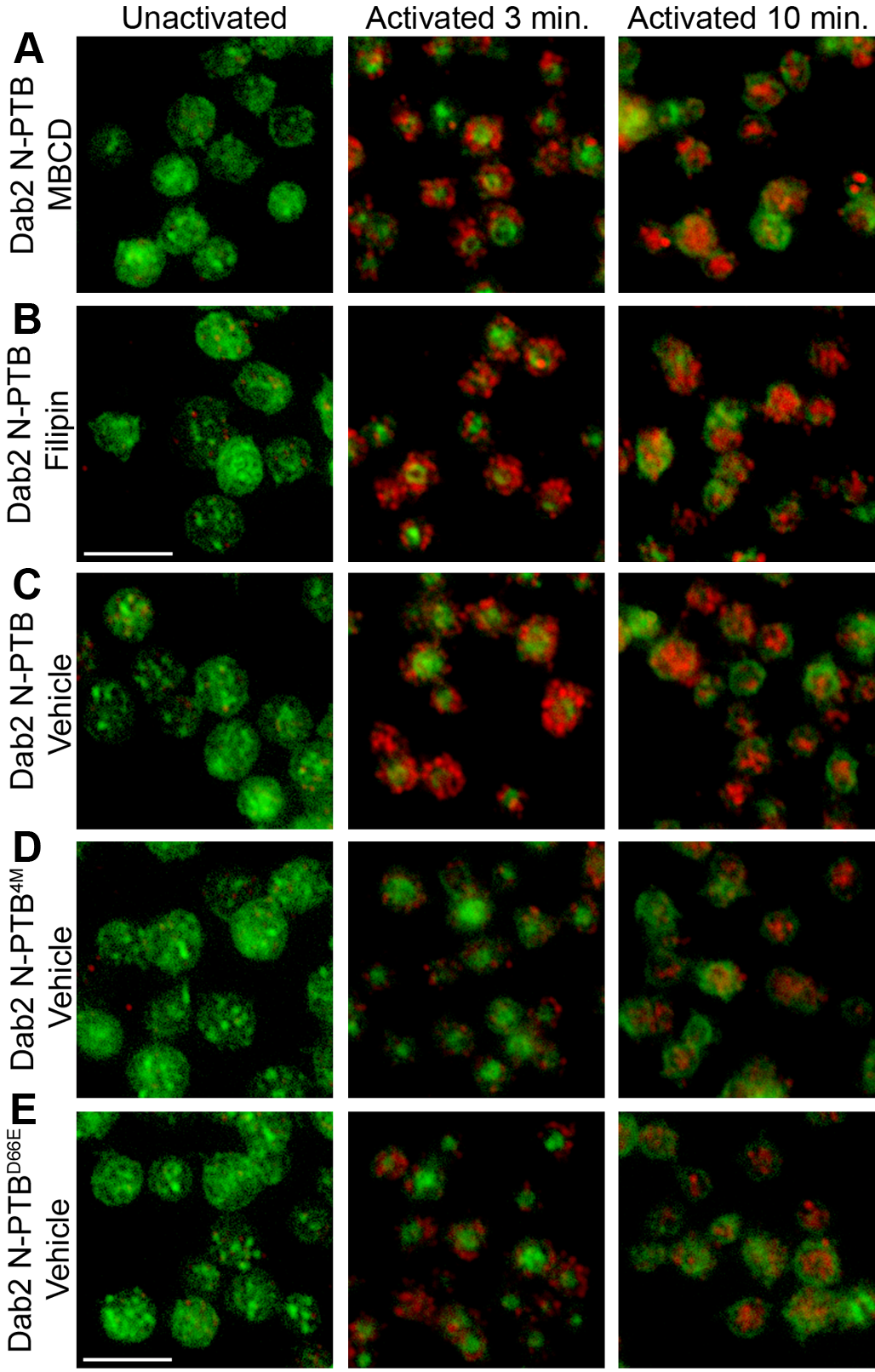

Supplement: Figure S6 — Analysis of platelet localization of the Dab2 N-PTB constructs using inhibitors of endocytosis. (A–B) Washed human platelets were pre-incubated with MBCD (A) or filipin (B) and further incubated with Dab2 N-PTB (1.9 µM) for 5 min at room temperature. Samples were fixed prior to activation (Unactivated), 3 min (Activated 3 min), and 10 min (Activated 10 min), after the addition of 10 µM TRAP and localization was monitored by immunofluorescence. (C–E) Vehicle controls for the chlorpromazine experiment shown in Figure 4F–H. Scale bar: 5 µm. The figure shows data from a single experiment that was repeated two times with similar results. (1.18 MB TIF) [file pone.0008007.s008.tif]
